# Joint reconstruction of neuron and ultrastructure via connectivity consensus in electron microscope volumes

**DOI:** 10.1186/s12859-022-04991-6

**Published:** 2022-10-31

**Authors:** Bei Hong, Jing Liu, Hao Zhai, Jiazheng Liu, Lijun Shen, Xi Chen, Qiwei Xie, Hua Han

**Affiliations:** 1 School of Artificial Intelligence, School of Future Technology, University of Chinese Academy of Sciences, Beijing, China; 2 National Laboratory of Pattern Recognition, Institute of Automation, Chinese Academy of Sciences, Beijing, China; 3 Research Base of Beijing Modern Manufacturing Development, Beijing University of Technology, Beijing, China; 4 CAS Center for Excellence in Brain Science and Intelligence Technology, Shanghai, China

**Keywords:** Connectomics, Reconstruction, Connectivity concept, Joint optimization, Electron microscope volumes

## Abstract

**Background:**

Nanoscale connectomics, which aims to map the fine connections between neurons with synaptic-level detail, has attracted increasing attention in recent years. Currently, the automated reconstruction algorithms in electron microscope volumes are in great demand. Most existing reconstruction methodologies for cellular and subcellular structures are independent, and exploring the inter-relationships between structures will contribute to image analysis. The primary goal of this research is to construct a joint optimization framework to improve the accuracy and efficiency of neural structure reconstruction algorithms.

**Results:**

In this investigation, we introduce the concept of connectivity consensus between cellular and subcellular structures based on biological domain knowledge for neural structure agglomeration problems. We propose a joint graph partitioning model for solving ultrastructural and neuronal connections to overcome the limitations of connectivity cues at different levels. The advantage of the optimization model is the simultaneous reconstruction of multiple structures in one optimization step. The experimental results on several public datasets demonstrate that the joint optimization model outperforms existing hierarchical agglomeration algorithms.

**Conclusions:**

We present a joint optimization model by connectivity consensus to solve the neural structure agglomeration problem and demonstrate its superiority to existing methods. The intention of introducing connectivity consensus between different structures is to build a suitable optimization model that makes the reconstruction goals more consistent with biological plausible and domain knowledge. This idea can inspire other researchers to optimize existing reconstruction algorithms and other areas of biological data analysis.

## Background

Connectomics, a concept proposed by Sporns [1], aims to comprehensively map the structure of neuronal networks in the nervous system to improve our understanding of how the brain works. Connectomics can be conducted at different spatial scales corresponding to the observational scale of brain imaging, these scales can be roughly divided into the microscale, mesoscale, and macroscale [2]. Nanoscale connectomics, which aims to map the fine connections between neurons with synaptic-level detail, has attracted widespread interest from researchers. Electron microscopy (EM) is currently the only imaging technique with the required synapse-scale resolution and the ability to obtain a sufficiently large data set to encompass a significant number of local neural circuits. Since Sydney began manual mapping of the complete connectome of C. *elegans*, an effort that lasted over a decade [3], the throughput of EM imaging has increased by several orders of magnitude [4, 5], substantially advancing the field of connectomics. Automated analysis of the EM data is crucial due to the vast data volume. However, the automation of image analysis is insufficient at this time. Currently, the main bottleneck is that automatic reconstruction results still require heavy manual editing [6–8]. The primary goal of this research is to improve the accuracy and efficiency of neural structure reconstruction algorithms.

**Fig. 1:**
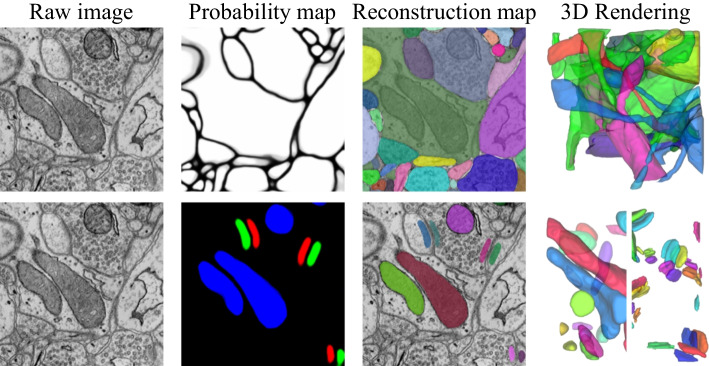
The representative pipeline for the image analysis in electron microscopy images. *Top* neuron reconstruction. *Bottom* ultrastructure reconstruction

As shown in Fig. 1, the goal of image analysis in connectomics is to reconstruct cellular and subcellular structures on three-dimensional (3D), i.e., the instance segmentation. The sparse distribution of subcellular structures, also known as ultrastructures, including synapses and mitochondria, reduces the reconstruction difficulty to some extent. Due to being well-isolated, most reconstruction algorithms adopt a bottom-up approach and emphasize the acquisition of semantic segmentation [9–13]. To obtain the reconstruction result, Li et al. [14] propose a coarse-to-fine 3D connection algorithm based on intersection-over-union (IoU) to assign the segmentation label. Two straightforward and efficient connection algorithms, a watershed-based method and connected component labeling, are introduced in MitoEM challenge [15]. Despite satisfactory results, the generalization ability of the above methods for complex cases remain unknown, especially in practical applications with random factors such as sample wrinkle and imaging damage.

Compared with ultrastructures, reconstructing neurons proved more challenging lie in: densely intertwined, irregularly-shaped and non-differential staining. Existing methods mainly distinguish them by membrane boundaries, but local minor detection errors may lead to severe merging errors that are time-consuming to correct. Therefore, the prevailing workflow typically consists of two stages [16–19]. Semantic segmentation of the membrane boundary is performed first, followed by generating over-segmented fragments and determining the connection relationships between the segments [17]. Membrane boundary detectors have demonstrated the ability to provide satisfactory results due to recent advances in convolutional neural network (CNN) [20–23]. Agglomeration algorithms for determining the connections between fragments have attracted extensive research. Most works perform greedy merging of segments based on the highest probability of similarity [18, 24–26]. Others regard the agglomeration as a graph partition problem with globally constrained mathematical properties [16, 27–30]. Another type of reconstruction algorithm called flood-filling networks [31], tracks from the seed points and uses a recurrent neural network to grow the neuron iteratively by moving the receptive field.

**Fig. 2:**
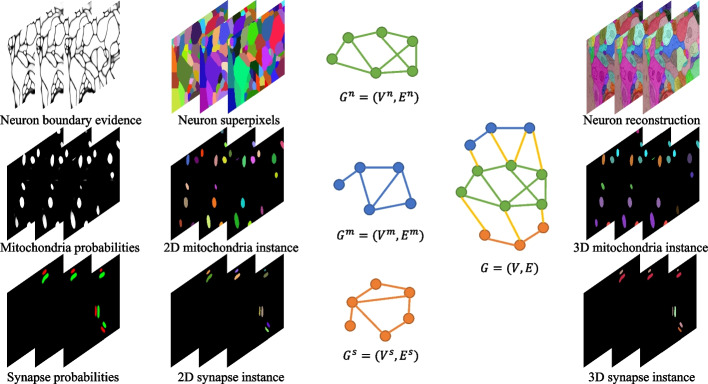
Overview of the proposed method. Most current reconstruction pipelines consist of two-step, ignoring the joint connectivity of the different hierarchies. We extend this pipeline by introducing the connectivity consensus to create an overall optimization framework. Consecutive steps from left to right: given the semantic input map of three structures, we perform segmentation to obtain the 2D instance and construct the graph. Then we combine the hierarchy graph and build the joint optimization model to obtain the connectivity and the reconstruction map simultaneously

We focus on the agglomeration algorithms in this paper. In this scope, the multicut algorithm [28] is widely used due to its clear mathematical formulation and graceful properties [16, 27]. As stated in [32], compared to other greedy algorithms [24], multicut brings better results due to the global objective and without external stopping criteria. On the other hand, since solving multicut is NP-hard, some works are optimized on local search algorithms in order to produce the high quality and fast solutions for large-volume data in the field of connectomics, including the greedy additive edge contraction (GAEC) solver, the Kernighan–Lin solver [33], the Fusion-Move solver [34], and the Block-Wise solver [35]. Agglomerative clustering with average linkage criteria (GASPavg) [25] and mutex watershed (MWS) [30] are signed graph partitioning algorithms and two well-known neuron agglomeration algorithms in connectomics. Specifically, GASPavg uses average linkage as the newly formed edge weight update criterion, replacing the sum linkage update criterion in GAEC, while MWS uses absolute maximum linkage. It is pointed out in [25] that different edge update criterion has a significant impact on the results.

Indeed, the neural structures are highly correlated. How to combine multi-level information in the reconstruction process has recently attracted great attention. In particular, Krasowski et al. [32] incorporate sparse biological priors and boundaries to extend the existing agglomeration algorithms. On this basis, Pape et al. [36] introduce a more general approach to leverage domain-specific knowledge to improve the segmentation result. Recently Wolf et al. [37] propose the semantic mutex watershed for joint graph partitioning and labeling. Out of connectomics, the correlation co-clustering (Co-Clustering) [38] utilizes both low-level and high-level cues to jointly address trajectory-level motion segmentation and multiple object tracking, which is similar to our methods. Levinkov et al. [39] define a combinatorial optimization problem, a general algorithm of lifted multicut algorithm that produces both decomposition and node labeling. Furthermore, several studies utilize prior knowledge in the post-processing step to improve existing results. Some publications underline the morphological characteristics of the reconstructed results [40–42], whereas others focused on sub-compartment category attributes [43] or specific labeled membrane data [44].

Nevertheless, the above approach retains a major drawback of a lack of joint connectivity of the different hierarchies. In other words, the reconstruction of each structure is independent and fails to form an overall optimization framework. As the most complex system known, neural structures do not exist in isolation, but have unique affiliations. Specifically, mitochondria, responsible for energy production, are commonly used to estimate the neuronal activity level, which always exists within a neuron. Meanwhile, synapses, which provide information transmission between neurons, quantify the neuronal connectivity strength. Since their advantages are complementary, it is reasonable and desirable to perform joint optimization of the reconstruction results based on different levels of connectivity priors.

To address this issue, we propose a joint optimization model for neural structure agglomeration in connectomics. The model can inherently integrate the connectivity consensus of both types of structures through constraints while yielding significant advantages in optimality (for an overview see Fig. 2). More precisely, the contributions of this paper are threefold:

- First, to the best of our knowledge, we introduce the concept of connectivity consensus and demonstrate its effectiveness for neural agglomeration in connectomics for the first time.
- Second, we propose a novel and bio-inspired graph partitioning model for joint optimization of neuron and ultrastructure reconstructions.
- Third, two structural linkage patterns, including mitochondria and synapses, are explicitly encoded and readily extended to other subcellular structures.

## Methods

### Motivation

**Fig. 3:**
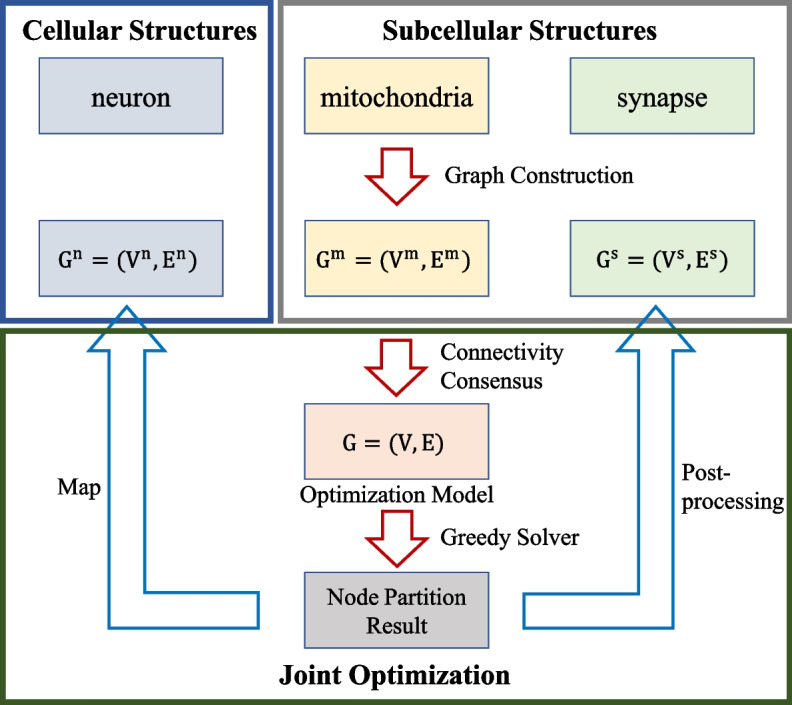
The flowchart of the method

Connectomics aims to reconstruct the fine connections between the neural structures of biological tissues. The image content is rich at the nanoscale, where fine subcellular structure such as mitochondria and synapse are clearly visible, while neurons are spread all around and distinguished by membrane boundaries (as shown in Fig. 1). Exploring the inter-relationships between structures will help EM image analysis. The core idea of this paper is to jointly optimize neuronal segments with boundary cues and ultrastructural regions with structural cues, which has the advantage of complementing the limitations of single connection cues. The flowchart of the method is shown in Fig. 3. The idea underlying joint optimization is that local single connectivity cues are not always reliable due to uncertainties, such as unclear imaging or poor alignment of EM images, whereas introducing inter-relationships can jointly optimize the reconstruction results by incorporating multiple cues.

### Connectivity consensus

For neural reconstruction, we introduce the concept of connectivity consensus between each structure. Specifically, mitochondria, as organelles of cellular energy supply, exist in the same cell. Therefore, beside their own connection relationship, the additional prior is that the 3D instance mitochondria are located in the same neuron. The synapse act as a hub for information transmission between different cells, where the pre-synapse is the axon terminal of the previous neuron and the post-synapse is usually the cell body or dendrite of the next neuron. Likewise, in addition to the connection relationship of the synapse itself, the extra connection information is that the neuron connected by the synapse comes from different instances. One of the benefits of connectivity consensus is that sparse ultrastructure connectivity is often more accurate than dense neuron structures.

**Fig. 4:**
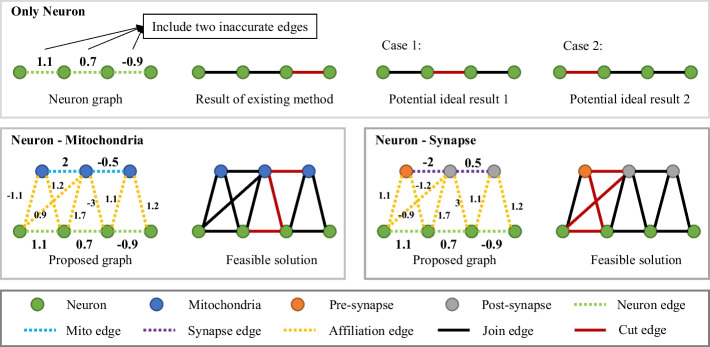
Two link patterns are defined according to the connectivity consensus. Neuron-Mitochondria reflects the link pattern between neuronal fragments and 2D mitochondria region. Neuron-Synapse represents the link pattern between the neuronal fragments and 2D pre- and post-synapse. Existing methods tend to be sensitive to inaccurate local edge weights, while the entire graph contains a richer global link- and not-link-information, allowing the feasible solution to minimize partitioning errors caused by inaccurate local edge weights

Based on the connectivity consensus, we define two link patterns shown in Fig. 4, to represent the neuron-mitochondria and neuron-synapse hierarchical relationships. Noted that, we categorize the synapses into pre-synapses and post-synapses according to [12], which is in line with the connectome as well as the proposed method (such as Fig. 1). In Fig. 4, green, blue, orange and grey circles indicate the neuron fragment, mitochondria, pre-synapse and post-synapse, respectively. The dashed green line indicates edges connecting two nodes between neuron fragments. The dashed yellow line indicates edges connecting the ultrastructure to the neuronal nodes. The dashed blue and purple lines indicate edges connecting two nodes between mitochondria and synapse, respectively. The connectivity consensus among the various structures implies that the final reconstruction results need to satisfy the connectivity consistency described above. As illustrated in Fig. 4, assuming that the three original neuron edges have two inaccurate edge weights (common but unknown in advance), then the existing method often fails to partition ideally due to the high reliance on local edge cues. On the contrary, the final graph partitioning result produced by our method still meets the plausibility expectation in this case, which is based on the advantage of the connectivity consensus among the structures. Although the forms of the two link patterns are similar, the final feasible solution differs significantly. The difference is that mitochondria bring link information, whereas synapses additionally contain not-link information. Note that although the blue circles both represent mitochondria, these may come from different neurons, which should be partitioned into two categories according to the connectivity consensus. Only when two mitochondria are located within the same neuron, that mitochondrion will be classified into one class.

### Optimization model

One of the contributions of this study is how to express the ultrastructures (mitochondria and synapse) and structures (neuron) hierarchies in EM images, enabling joint optimization of different neural structures. Based on connectivity consensus, we assume that the graph composed of neurons, mitochondria and synapses is defined as $G^{n}=(V^{n}, E^{n})$, $G^{m}=(V^{m}, E^{m})$ and $G^{s}=(V^{s}, E^{s})$, respectively, where $V^{n}$ is the set of nodes composed of neuronal fragments, and $V^{m}$ and $V^{s}$ are the segmentation regions of the mitochondria and synapse, respectively. The set of edges indicates that two connected nodes have the potential to belong to the same instance. Each edges with a cost $w \in R$ reflects its strength. The detailed graph construction is described below.

We further define the entire graph $G=(V, E)$ containing above structures whose $V= V^{n}\cup V^{m}\cup V^{s}$ and $E= E^{n}\cup E^{m}\cup E^{s} \cup E^{a}$, where $E^{a}$ is defined as an additional edge set $\left\{ u,v \right\} \in E^a$ that connects a neuron fragment $u\in V^{n}$ with an ultrastructure region $v\in V^{m}\cup V^{s}$, indicating the affiliation between them, i.e., belonging to the same object. Note that we try to construct a joint optimization model that obtains the connectivity relationships of both structures through a single once optimization. The optimization problem is expressed as an integer linear programming problem as follows to integrate the connectivity consensus and the hierarchy graphs:

1 $$\begin{aligned}&\min _{x\in \left\{ 0,1 \right\} ^{E } }\lambda _{n} \sum _{e^{n}\in {E^{n}} }w_{e^{n}}x_{e^{n}}+\lambda _{m}\sum _{e^{m}\in {E^{m}} }w_{e^{m}}x_{e^{m}} \nonumber \\&+\lambda _{s}\sum _{e^{s}\in {E^{s}} }w_{e^{s}}x_{e^{s}}+\lambda _{a}\sum _{e^{a}\in {E^{a}} }w_{e^{a}}x_{e^{a}}, \end{aligned}$$

2 $$\begin{aligned}&\text{ s.t. }\quad \forall Y\in {\rm{cycles}}(G),~\forall e\in Y:x_{e} \le \sum _{e^{'}\in {Y\setminus {\left\{ e \right\} } } }x_{e^{'}}, \end{aligned}$$

3 $$\begin{aligned}&\lambda _{n}+\lambda _{m}+\lambda _{s}+\lambda _{a}=1, \end{aligned}$$

where $x_{e}$ represents the binary indicator variable of each edge $e\in E$ in the final partition (1 is cut and 0 is join), and the weight $w_{e}\in R$ corresponds to each edge is the cost reflecting the attractive or repulsive strength. The objective function (2) is composed of four parts: the graph of neuron fragments, mitochondria and synapses, and the affiliation edges connecting them. $\lambda _{n},\lambda _{m},\lambda _{s},\lambda _{a}\in [0,1]$ are hyper-parameters derived from the domain knowledge to balance the reconstruction confidence level. ${\rm{cycles}}(G)$ denotes the set of all cycles in *G*. Inequality (2) constraint a consistent solution without “dangling edges”, i.e., a valid partition [45]. The feasible solution of the optimization problem is a decomposition of the graph *G*, where each component in the final solution represents the same class.

The primary difference that distinguishes the proposed method from previous approaches, such as asymmetric cuts [32, 46], correlation co-clustering [38] and semantic mutex watershed [37], is the proposed model considers the connectivity of the ultrastructures themselves, i.e., edge connections are added between the nodes of the ultrastructures. This set has two advantages, one is to prevent errors in node assignment, and the other is to overcome the limitations of a single connectivity. In other words, we overcome the fact that the neuron reconstruction relies too much on local boundary cues, while the ultrastructure reconstruction relies too much on local spatial information.

### Graph construction method

**Fig. 5:**
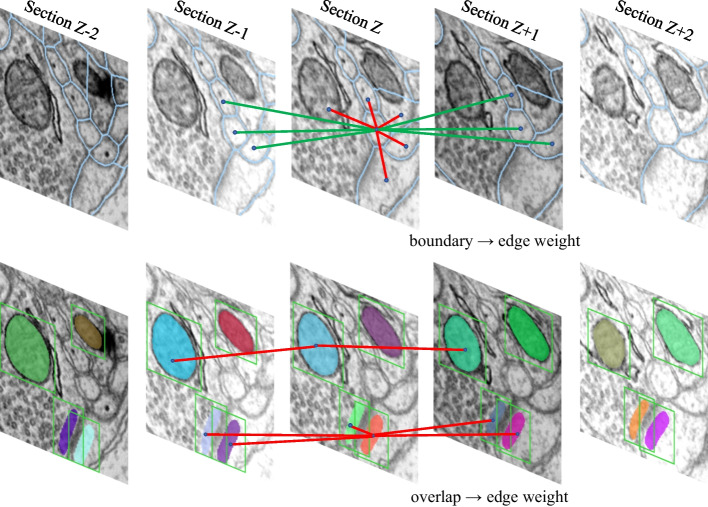
Examples of graph construction. *Top* neuron graph neighborhood of a single node with local plane-edges (red lines) and cutting-edges (green lines), the edge weight mainly derived from the membrane boundaries. *Bottom* example of the ultrastructure graph, nodes are ultrastructural regions, edges are represented by red lines, and the edge weight derived from their segmentation overlap

This section describes the construction of the graphs $G^{n}=(V^{n}, E^{n})$, $G^{m}=(V^{m}, E^{m})$ and $G^{s}=(V^{s}, E^{s})$ and the definition of the affiliation edge set $E^{a}$. Figure 5 shows examples of the graph construction.

#### The construction method of neuron graph

The graph $G^{n}=(V^{n}, E^{n})$ is defined as the region adjacency graph (RAG) composed of the superpixels of neurons, where *V* is the set of neuronal fragments and $E\subset V\times V$ represents the set of edges connecting any adjacent nodes. For each edge $e^n :=\left\{ u,v \right\} \in E^n$, a weight $w_{e^n}\in R$ is assigned to represent the similar strength of fragments *u* and *v*, which is usually obtained from the similarity $p_{e^n}\in [0,1]$ via negative log-likelihood function, defined as:

4 $$\begin{aligned} w_{e^n}=\log \frac{1-p_{e^n}}{p_{e^n}}+\log \frac{1-\beta }{\beta }, \end{aligned}$$

where the bias hyper-parameter $\beta \in [0,1]$ controls the degree of over-segmentation. If $\beta > 0.5$, then the edge weight $w_{e^n}$ decreases, otherwise the $w_{e^n}$ increases. In terms of object function (2), if the edge weights tend to be negative, it means that the stronger repulsive strength of nodes *u* and *v* corresponds to over-segmentation. Conversely, it means under-segmentation. Usually, $\beta$ takes a default value of 0.5, which is adjusted according to the similarity $p_{e^n}$ correspondingly.

Generally, there are two ways to calculate the similarity $p_{e^n}$. It can be obtained from the mean affinity of the membrane prediction, with a assumption that the quality of convolutional network output is satisfied [26]. The other more complex method requires multiple steps [36]. First, several descriptions of each edge are extracted from the raw images, boundary predictions and corresponding filtered images (including gaussian filter, hessian filter and laplacian filter). Specifically, the feature set is the same as described in [16], where the extracted features include boundary appearance feature, region statistical feature and shape topology feature. Subsequently, the extracted feature vectors are fed into the classifier to predict whether the contact between two superpixels represents same neuron or not. The difference between these two methods is that the similarity after classifier relearning is more accurate and reliable than the mean affinity value. However, the latter requires more time for feature extraction, and is more suitable for data with low cutting-axis continuity.

#### The construction method of ultrastructure graph

The definition of ultrastructure graph should consider the connectivity consensus; otherwise, additional errors may be introduced, leading to model failure. Figuratively, a cell may contain several mitochondria, i.e., it is impossible to determine whether the connectivity between different mitochondria is consistent with the connectivity between neuronal fragments. Since the 3D image stacks are aligned, we design a method for calculating the edge weights according to strong context clues. Specifically, for graph $G^{m}=(V^{m}, E^{m})$, we only add edges that potentially belong to the same instances of mitochondria. For graph $G^{s}=(V^{s}, E^{s})$, we expect the edges between the pre- or post-synapses to be repulsive, whereas the edges within the pre- or post-synapses are attractive.

For present, we take $G^{m}$ as an example, where synapse graph $G^{s}$ is built in a similar way to mitochondria. Formula, we assume image volumes has *M* sequential slices. For every pair of segmentation region $u,v\in V^{m}$ in $M_u$ slice and $M_v$ slice with $\left| M_u-M_v \right| \le 2$, we define the probability $p_{uv}\in [0,1]$ of their plain bounding boxes $d_u, d_v$ IoU and segmentation area $s_u, s_v$ overlapping belonging to the same object as:

5 $$\begin{aligned} p_{uv} = \frac{D_{uv}+\lambda S_{uv}}{1+\lambda }, \end{aligned}$$

where $D_{uv}=\frac{d_u\cap d_v}{d_u\cup d_v}$ measures the similarity of the spatial position, $S_{uv}=\frac{s_u\cap s_v}{s_u\cup s_v}$ characterizes the invariance of the segment shape, $\lambda \ge 0$ is a regularization parameter to balance the coefficients of the boxes and segment. We define the edge set as $E^{m}: =\left\{ (u,v)~|~p_{uv}> 0, \left| M_u-M_v \right| \le 2 \right\}$, which means that only ultrastructures in close contact are considered as potential links. Then, for each edge $e^m :=\left\{ u,v \right\} \in E^m$, the edge cost $w_{e^m}$ is derived from $p_{uv}$ via negative log-likelihood function, similar to Eq. (4). The default set of the bias hyper-parameter $\beta$ is 0.5. If the image volumes is highly anisotropic, then $\beta$ decreases. Note that more sophisticated methods have been proposed to characterize the ultrastructural similarity [14], but they have higher computational complexity. The experimental results show that the proposed similarity formula is sufficient to bring about performance improvements due to the constraints of the other edges.

#### The construction method of affiliation edges

For every neuronal segments $u\in V^n$ and every ultrastructures region $v\in V^m\cup V^s$, we define the affiliation edge set $E^a$ based on the maximum overlap area as:

6 $$\begin{aligned} E^{a}: =\left\{ (u,v)~|~A(s_u\cap s_v)> A_t \right\} , \end{aligned}$$

where $A(\cdot )$ denote the pixels numbers of overlap area, $A_t$ represent the area threshold. Regarding affiliation edge definition, we only retain edges with a certain confidence level once the maximum overlap area exceeds $A_t$. Since this step may filter out some small regions, we believe it can reduce the invalid information resulting from detection errors, which is important for graph construction. In another aspect, it can decrease the model’s complexity because it contains fewer nodes and edges.

Assume $P^i|_{i=1}^{M}$ is the ultrastructure probability map sequences. We calculate the probability $p_{uv}\in [0,1]$ reflecting the strength of the affiliation as:

7 $$\begin{aligned} p_{uv}=\sum _{j\in t}{P_{j}^{M_v}}, \end{aligned}$$

where *t* is the overlap regions and $M_v$ denote the corresponding probability map sequence. That is, the probability $p_{uv}$ is defined as mean response of network output in *t*. Then for each edge $e^a :=\left\{ u,v \right\} \in E^a$, the edge weight $w_{e^a}$ is also transformed from $p_{uv}$ by Eq. (4). Similarly, the default bias parameter $\beta$ depends on the segmentation performance of the ultrastructure and has a default value of 0.5. Intuitively, if the segmentation quality of the ultrastructure is satisfactory, then $\beta > 0.5$; otherwise, $\beta < 0.5$. Notice that all above parameters are hyper-parameters, which can be verified through a grid search in the validation dataset.

### Greedy solver

After the above steps, we have constructed multiple undirected graphs and established an optimization problem, the next step is to solve the model. Since cycle constraints increases exponentially with the number of nodes, finding the optimal solution of the optimization problem is NP-hard. Most existing solvers use greedy heuristic algorithms to generate feasible solutions with good results, although establish neither lower bounds nor approximation certificates [33, 35]. In this paper, we employ the greedy additive edge contraction (GAEC) solver [29], which is a fast local search heuristic, although it operates directly on the nodes without any local pre-clustering. This solver runs only once to monotonically produce deterministic feasible solutions, thus providing low computational cost even for large-volume data. GAEC always makes the greedy choice that most decreases the objective function (2). Specifically, GAEC merges the edges with the highest weights in each loop, contracts the edges and shrinks the graph, and re-computes the cost of the changed edges with sum linkage. These steps are repeated until all edges in the contracted graph have a negative value, then the algorithm terminates. We implement this procedure by query sorting, and the time complexity of the solution is $O(V^2\log V)$.

### Post-processing method

After obtaining the edge state in the entire graph *G*, we assign a unique label to each connected component. All nodes of each component are assigned the same label. We map the node label to the subgraph $G^n$, $G^m$, and $G^s$ correspondingly. The proposed model may partition different instances of the ultrastructure into the same category according to the high-level prior information of the neurons, which facilitates the detection of specific neural structures such as multi-contact synapses [47]. Since others research focus on 3D instances of the ultrastructure itself, separating incorrectly merged ultrastructures is necessary for the final reconstruction results in this case. We design a simple post-processing strategy to distinguish ultrastructures without edge connections in graph $G^m$ or $G^s$. Specifically, if two nodes in $G^m$ are in the same partition in the final solution of *G*, but there are no paths connecting them in graph $G^m$, a new label is assigned to one of the components. This procedure is iterated until there are no more violations of the node assignments. Using this approach, incorrectly merged ultrastructure can be effectively partitioned into several classes based on primary edge priors.

## Experiments and results

### Datasets

We use the Harris dataset and Snemi dataset for comparison, as described below:

*Harris dataset* comes from the hippocampus of adult rats with a resolution of $2\times 2\times 50~nm^{3}$, including the apical dendrite dataset and the spine dataset [48]. Due to the background region and incomplete annotation, we only select the region with neuron groundtruth labeling to facilitate the evaluation of algorithm performance. For apical dendrite dataset, we cut a subvolume of $1536 \times 1536 \times 100$ voxels , and divided the data into train set (sections 1-50) and test set (sections 51-100). For spine dataset, we cut a subvolume of $904\times 865 \times 42$ voxels , and divided the data into train set (sections 1–21) and test set (sections 22–42).

*Snemi dataset* is created by Kasthuri et al. [7] and contains labeled subvolumes from the mouse somatosensory cortex. The dataset contains 100 consecutive slices for training and 100 slices for testing. Each slice has a size of $1024\times 1024$ and a resolution of $6\times 6\times 30~nm^{3}$.

### Error metrics

Two common error metrics are used to evaluate the reconstruction performance: variation of information (VI) [49] and adapted rand error (ARE) [50]. A lower value corresponds to higher segmentation quality in both metrics. The background was ignored when evaluating. The expression of the VI is as follows:

8 $$\begin{aligned} VI(S,T) = H(S|T) + H(T|S), \end{aligned}$$

where *S* and *T* are the automatic reconstruction and gold standard respectively, $H(\cdot )$ represents the conditional entropy, and *H*(*S*|*T*) and *H*(*T*|*S*) quantify over- and under-segmentation, respectively.

### Baseline methods

We evaluate the reconstruction metrics from two aspects. For comparing the neuron reconstruction results, we adopt six representative methods with the same input data and graphs, including:

- *MC* the standard multicut algorithm with same GAEC solver [28] is used as a baseline, it is a well-known agglomeration algorithm without needing to specify the number of clusters, but relies only on boundary cues.
- *GASPavg* agglomerative clustering with average linkage criteria [25] use average linkage as the newly formed edge weight update criterion, which is a more robust method of updating edge weights for inaccurate edge.
- *MWS* mutex watershed [30] is an efficient algorithm for signed graph partitioning, it adopt absolute maximum linkage by encode both attractive and repulsive cues with nearly linearithmic complexity.
- *MC w/ KL* the standard multicut algorithm with Kernighan-Lin solver [33], where Kernighan-Lin solver starts from any initial cluster and transforms the nodes in each iteration to reduce the decomposition cost.
- *MC w/ FM* the standard multicut algorithm with Fusion-Move solver [34], where Fusion-Move solver iteratively fuses the current and the proposal solutions and empirically produces lower objective function for multicut problem.
- *Co-Clustering* correlation co-clustering [38], a model that can combine multi-level information but ignore the domain knowledge.

All methods have been used for the partition of signed graphs [27–29, 33, 34] and have been widely used in neuron reconstruction [16, 25, 30]. For the proposed method (Ours), we report two versions that without mitochondria information (Ours w/o mito) and without synapse information (Ours w/o syn), to demonstrate the effectiveness of integrating various connectivity relationships.

For comparing the ultrastructure reconstruction results, we employ the popular 3D connected component labeling (CC labeling) [15]. In particular, we propose a baseline (Ours w/o neuron), i.e., when $\lambda _{n}, \lambda _{a}=0$ in Eq. (2) and $G^{n}=\emptyset$, this objective function can be treated as a special case to solve only the partition problem of the ultrastructure.

**Fig. 6:**
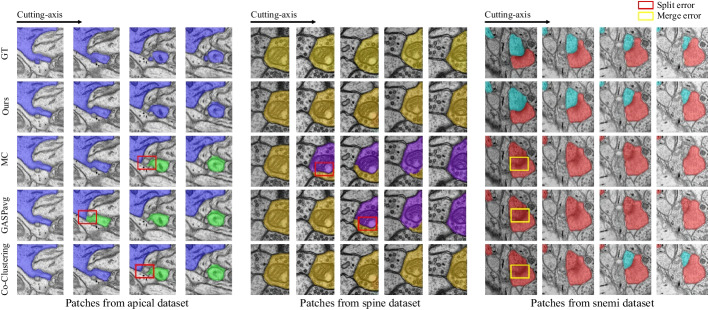
Qualitative comparison of the neuron reconstruction performance from serial patches on two datasets. *Left* and *Middle* patches from Harris dataset. *Right*: patches from Snemi dataset. The first column displays the groundtruth of the neuron. The last four columns show the results generated by Ours, MC, GASPavg, and Co-Clustering, respectively. The split errors are highlighted in the red boxes, and the merge errors are highlighted in the yellow boxes

**Fig. 7:**
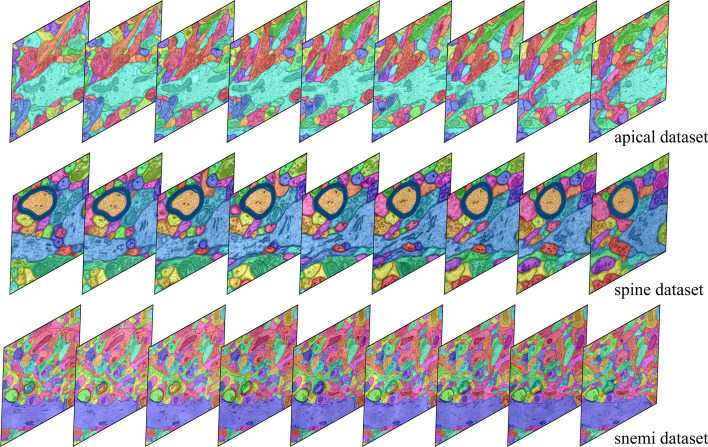
Visualization of the proposed method on two datasets respectively

**Table 1:** The quantization performance of the neuron reconstruction on Harris dataset

Methods	Apical				Spine			
	VI	VI-Split	VI-Merge	ARE	VI	VI-Split	VI-Merge	ARE
MC	0.7373	1.0487	0.4259	0.1431	0.4329	0.6218	0.2439	0.1087
GASPavg	0.7320	1.0489	**0.4152**	**0.1389**	0.4199	0.6067	**0.2330**	0.1067
MWS	0.7598	1.0780	0.4415	0.1460	0.4364	0.6252	0.2477	0.1111
MC w/ FM	0.7758	1.0797	0.4719	0.1594	0.4645	0.6760	0.2530	0.1228
MC w/ KL	0.7753	1.0741	0.4766	0.1657	0.4156	0.5915	0.2396	0.1058
Co-Clustering	0.7374	1.0498	0.4249	0.1430	0.4094	0.5746	0.2441	0.1037
Ours w/o mito	0.7329	1.0459	0.4198	0.1427	0.4230	0.6021	0.2440	0.1061
Ours w/o syn	0.7319	1.0439	0.4199	0.1418	**0.4084**	**0.5726**	0.2442	**0.1035**
Ours	**0.7307**	**1.0421**	0.4192	0.1418	**0.4084**	**0.5726**	0.2442	**0.1035**

**Table 2:** Comparison of neuron reconstruction performance with and without ultrastructural mapping regions on Harris dataset

Apical						Spine					
	Methods	VI	Decrease	ARE	Decrease		Methods	VI	Decrease	ARE	Decrease
**w/**	MC	**0.2926**		**0.0708**		**w/**	MC	0.2139		0.0592	
	Co-Clustering	0.2969	+$1.47\%$	0.0709	+$0.14\%$		Co-Clustering	0.1776	−$16.97\%$	0.0531	$-10.30\%$
	Ours	**0.2926**	$0\%$	**0.0708**	$0\%$		Ours	**0.1758**	−$17.81\%$	**0.0527**	$-10.98\%$
**w/o**	MC	0.9359		0.3006		**w/o**	MC	0.5133		0.1487	
	Co-Clustering	0.9329	−$0.32\%$	0.2996	$-0.33\%$		Co-Clustering	0.5030	−$2.01\%$	0.1470	$-1.14\%$
	Ours	**0.9254**	−$1.12\%$	**0.2964**	$-1.40\%$		Ours	**0.5025**	$-2.10\%$	**0.1469**	$-1.21\%$

**Fig. 8:**
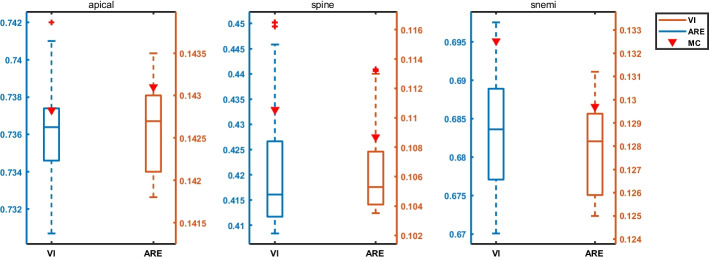
Robustness results for different confidence parameters between structures. The results of the error metrics are shown for the MC baseline (triangles) and the proposed method. Note that lower VI (blue) and ARE (red) are better

**Table 3:** The quantization performance of the ultrastructure reconstruction on Snemi dataset and Harris dataset

Dataset	Methods	Mitochondria				**Synapse**			
		VI	VI-Split	VI-Merge	ARE	VI	VI-Split	VI-Merge	ARE
Apical	CC labeling	0.0424	0.0076	0.0771	0.0382	0.0236	0.0468	0.0003	0.0065
	Ours w/o neuron	0.0044	0.0076	0.0012	0.0003	0.0207	0.0411	0.0003	0.0044
	Ours	**0.0044**	0.0076	0.0012	0.0003	**0.0014**	**0.0024**	0.0003	**0.0001**
Spine	CC labeling	0.0012	0.0025	0	0	0.0454	0.0908	0	0.0061
	Ours w/o neuron	0.0012	0.0025	0	0	0.0454	0.0908	0	0.0061
	Ours	0.0012	0.0025	0	0	** 0.0233**	**0.0467**	0	**0.0031**
Snemi	CC labeling	0.2387	0.2220	0.2554	0.0695	1.4642	0.6634	**2.2651**	0.9134
	Ours w/o neuron	0.2472	0.2390	0.2554	0.0728	1.4734	0.6816	2.2652	0.9184
	Ours	**0.2385**	**0.2218**	0.2554	0.0695	**1.4608**	** 0.6472**	2.2745	**0.9129**

### Experiments on Harris dataset

#### Experimental setting

In this experiment, we investigate the effect of the proposed model on Harris dataset. The semantic maps of neurons, mitochondria and synapses as a prerequisite to apply the joint optimization model of the neural structure. We train a 3D U-net for membrane/non-membrane pixel-wise semantic segmentation, and use manually annotated masks as ultrastructural semantic maps on Harris dataset. The network architecture is similar to [26], which differs from the standard 3D U-net in three ways: first, the residual module is employed to improve the model network representation; second, the feature maps of different levels are fused by element-wise summation operation, replacing the concatenation operation; third, the network never downsamples the z-axis resolution, in order to adapt to anisotropic data. For the neuron membrane, the output is nearest neighbor affinities as described in [18, 51]. We use binary cross-entropy as the network loss function, and adopt the Adam optimizer for stochastic optimization. The obtained probability map is then smoothed by a Gaussian function, where the local minima serve as seeds for the watershed algorithm to generate the superpixels of the neuron over-segmented fragments. Due to the anisotropy of the data, the superpixels are generated by each 2D image in the stack singularly. From above steps, we set up the optimization problem as follows: we build the region adjacency graph $G^n$ from the superpixels, that is, edges are also introduced between superpixels in adjacent slices. We train two random forest classifier to calculate the edge weight based on edge and region appearance features following [16]. One of the classifier learn the plane-axis edges (edges between superpixels in same slices) and the other learn the cutting-axis edges (edges between superpixels in adjacent slices), shown in Fig. 5. Since the classifier is supervised learning, to obtain the edge labels required for the training process, the edge is labeled as 0 if the two regions connected by this edge in groundtruth belong to the same neuron, otherwise labeled as 1. Then the features and the corresponding labels are fed into the random forest classifiers. The hyper-parameter of classifiers can be determined by classification accuracy via grid research. After training, those random forest classifiers are used to infer the similarities $p_{e^n}$ in test dataset.

Since this dataset contains few ultrastructures, we use ImageJ software [52] to annotate the binary mask in order to clarify the effect of the the joint optimization idea. We annotate 45 mitochondria and 50 synapses in the apical dataset, and 12 mitochondria and 8 synapses in the spine dataset. We use 2D connected component labeling of the binary mask to obtain the ultrastructure node set. Follow, the graphs $G^m$ and $G^s$ are constructed. The affiliation edge set $E^a$ is derived from the binary mask with an area threshold $A_t = 50$.

#### Results

We compare the neuron reconstruction results and ultrastructure reconstruction results separately. We record the quantitative analysis of neuron reconstruction in Table 1. The best result are highlighted in bold. Overall, introducing additional ultrastructural connectivity constraints show a clear improvement in performance over the previous method. Specifically, our method outperforms the standard MC by $0.90\%$ (for VI metric) on apical dataset (0.7307 versus 0.7373) and by $5.66\%$ on spine dataset (0.4084 versus 0.4329). Compared with other popular signed graph optimization strategies, our method performs better in most cases. Although the KL and FM approximate solvers empirically yield lower objective function solutions in multicut problem, this outcome does not always correspond to better reconstruction performance for the neuron task, e.g., for the apical dataset. The main reason may be the inaccurate edge weights since existing methods are sensitive to edge weights. In the spine dataset, the VI of the KL solver is lower than that of the GAEC solver (0.4156 versus 0.4329), but our model can compensate for the discrepancy caused by the approximate solution error and produces better reconstruction performance. The results of ablation studies integrating different structures show that neuron reconstruction performance benefits continuously with the introduction of synapses, mitochondria and full structure. Notice that the result of Ours w/o syn and Ours are consistent in the spine dataset, which may be attributed to two factors: (1) different ultrastructure may be related to the same neuron nodes resulting in the same performance gains, and (2) the edge weights of the synapses in this dataset fail to accurately reflect the connection strength. Unlike the Co-Clustering, the proposed model adds confidence factor derived from domain knowledge to balance the connection strength of each hierarchy. The error metrics confirm that the confidence factor is beneficial to the reconstruction results.

As intuitively shown in Fig. 6, we further qualitatively analyze in which cases our model brings benefits in neuron reconstruction. We observe that ours model can effectively reduce connection errors than the other methods. Observing patches from the spine dataset, it makes sense that the structural information of the mitochondria prevents split errors. While observing patches from the apical dataset, although this neuron does not contain ultrastructure, the nearby mitochondria are constrained in the optimization model, resulting in a reasonable overall result. More examples of neuron segmentation are given in Fig. 7, where each row shows the visualization results of the proposed method in consecutive 9-layer slices. Table 2 illustrates the algorithm advantages, where the metric improvement is measured separately in regions with/without the ultrastructure. The joint optimization model contributes to significant improvements over the baseline in regions with ultrastructure, whereas the other regions exhibit limited improvement due to global optimization. What’s more, the robustness results of the model to hyper-parameters on the Harris dataset are shown in Fig. 8. We record the VI and ARE of the proposed method with MC under different parameters. In most cases, the proposed model outperforms the baseline model.

The results of ultrastructure under the same hyper-parameter are shown in Table 3. Note that we used manually labeled ultrastructural data based on the 2D binary mask information, and our goal is to obtain the 3D label, which is not against our original intention. All error metrics are low because only connection errors exist in the reconstruction results. The result of CC labeling is close to the proposed method, but prone to introduce merge errors for the mitochondria and split errors for the synapse, as indicated by VI-Merge and VI-Split in Table 3. The reason is that CC labeling is more sensitive to changes in the cutting-axis and morphology because it determines whether the binary regions are the same instance by 26-neighborhood connectivity. By the way, the performance of the proposed baseline (i.e. Ours w/o neuron) is comparable to that of the joint optimization (i.e. Ours).

### Experiments on the Snemi dataset

#### Experimental setting

This experiment investigate the robustness and practicability of the proposed method under automatic ultrastructure segmentation algorithm. Similar to Harris dataset, we use the same method to generate the superpixels and RAG $G^n$. The neuronal fragment probability $p_{e^n}$ is the mean affinity value derived from the network output. For the ultrastructure, we utilize the same network architecture as in membrane segmentation to obtain the probability result, except for slight differences in the settings. For the mitochondria, we add the 2D instance contour as the target output to distinguish the planar instance results, refer to [15]. For the synapse, we use a model with the target of three channels to train a semantic segmentation network, refer to [12]. The three channels include pre-synaptic region, post-synaptic region, and synaptic region (union of the first two channels). After obtaining the semantic map, we transform them into binary images by using the binarization threshold, and obtain the initial segmentation result using the 2D connected component labeling. In this way, we can obtain pre/post synapses. We present the The input size of all the above networks is $256\times 256\times 8$, the optimizer is Adam, the training phase uses sufficient data augmentation to improve the generalization performance, and the model is iterated 20,000 times under 2 NVIDIA Tesla V100 GPUs with a batch of 8. The graphs $G^m$ and $G^s$ are similar to those in experiments on the Harris dataset, except that the binary mask is generated by binarization of the network output. The construction of the affiliation edges $E^a$ was conducted with an area threshold of $A_t = 50$.

#### Results

**Fig. 9:**
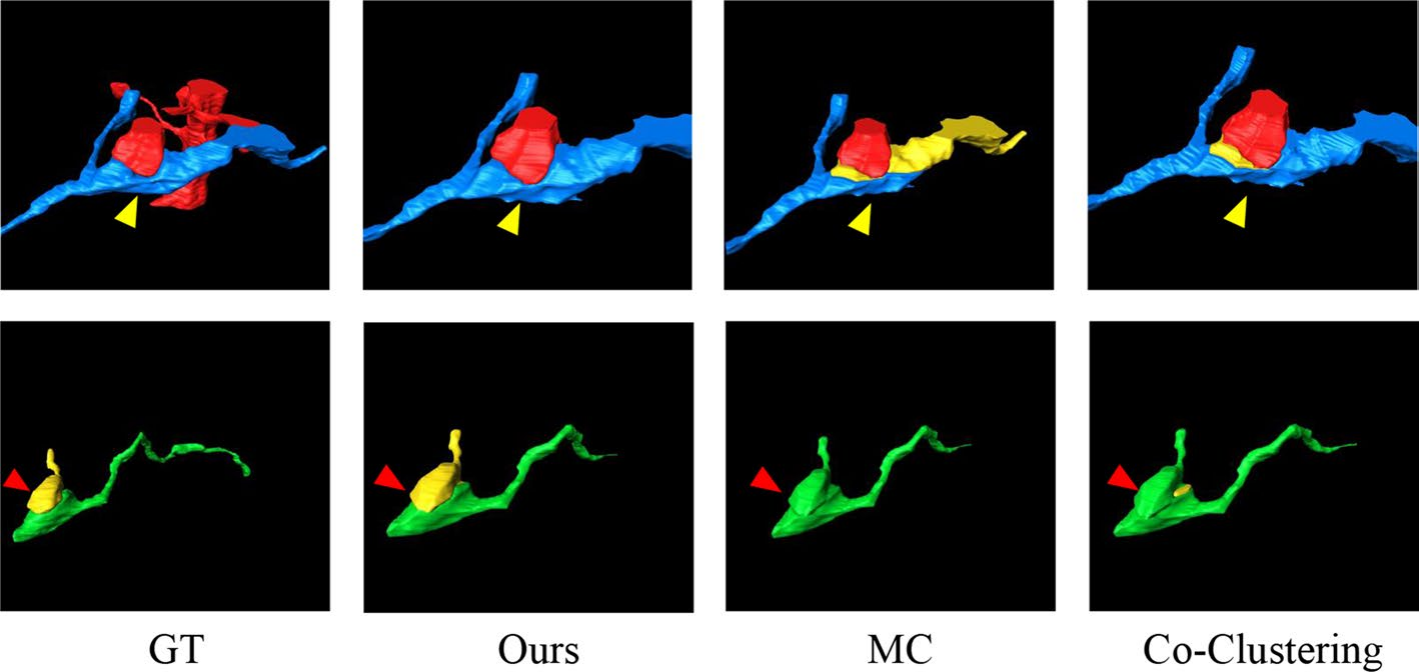
Quantitative comparison of two neurons with synaptic connections on Snemi dataset. Yellow and red arrows indicate major differences

**Fig. 10:**
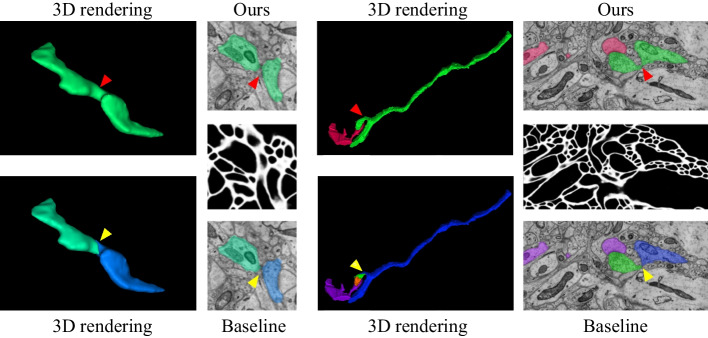
Examples of 3D renderings on Kasthuri dataset. *Left*: comparison of a single neuron with mitochondria. *Buttom*: comparison of two neurons at synaptic connections. Yellow and red arrows indicate major differences

We summarize three experiments of neuron reconstruction in Table 4 , including (i) the performance of the six representative methods, (ii) the results of the ablation studies on different ultrastructural connectivity constraints, (iii) the robustness of the potential improvements. Table 4 indicates differences in the neuron reconstruction performance of the six unsigned graph agglomeration methods. Particularly, MWS has the lowest time complexity $O(V\log V)$ but the results are not ideal. GASPavg, which adopts the new edge update criteria to increase the robustness of the weights, provides a good performance unsurprisingly. Compared to baseline MC, our approach integrates synapses, mitochondria and full information to decrease VI metrics by $1.25$, $2.79$ and $3.60\%$ respectively, demonstrating the effectiveness of joint connectivity cues. Compared to baseline MC, the method of Ours w/o mito, Ours w/o syn and Ours VI metrics lower $1.25$, $2.79$ and $3.60\%$ respectively, demonstrating the effectiveness of joint connectivity cues. Furthermore, as shown in Table 5, the VI of our method that considers only the fragments containing ultrastructures is $9.70\%$ lower than that of the baseline. Ours also outperforms the Co-Clustering, which only $6.54\%$ lower than that of the baseline. The qualitative comparison of the reconstruction results in Fig. 6 illustrates the main performance gains of the proposed method. Observation of the patches from Snemi dataset, we find that not-link-information of the synapses reduces the merge error, demonstrating the superiority of our method. The neuron segmentation performance of the proposed method for consecutive 9-layer slices on the Snemi dataset can be seen in Fig. 7. The 3D rendering results of the different algorithms at the synaptic connections of the two neurons are depicted in Fig. 9, which intuitively indicates that our method is the closest to the groundtruth. More 3D rendering example of the other regions in Kasthuri dataset [7] are available in Fig. 10. We also compare the error metric of our method with different confidence factors (with steps of 0.05) as shown in Fig. 8. Our method results in lower errors than the baseline in most cases, indicating the robustness of the proposed method.

**Fig. 11:**
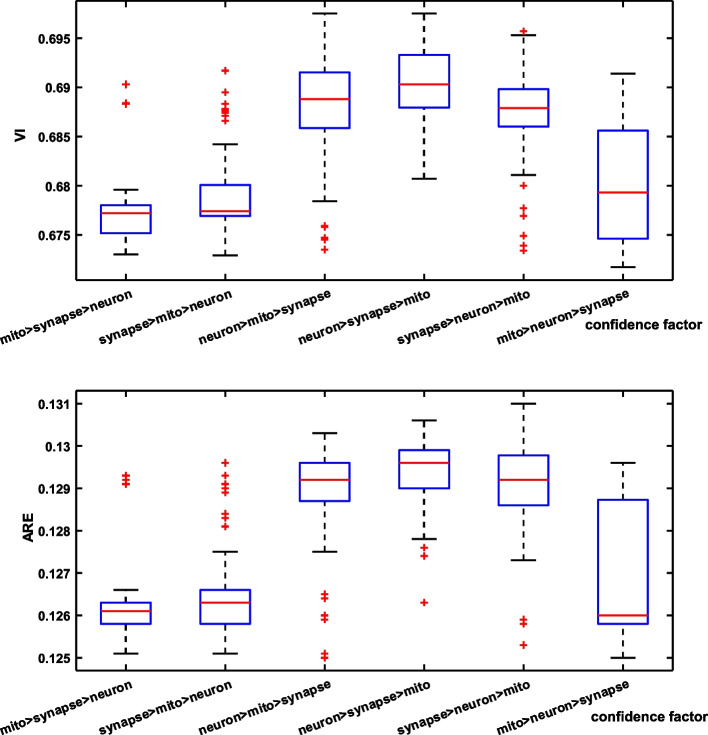
Comparison of the neuron reconstruction performance for three structures with different confidence factors on Snemi dataset. *Top*: the VI metric. *Down*: the ARE metric

**Fig. 12:**
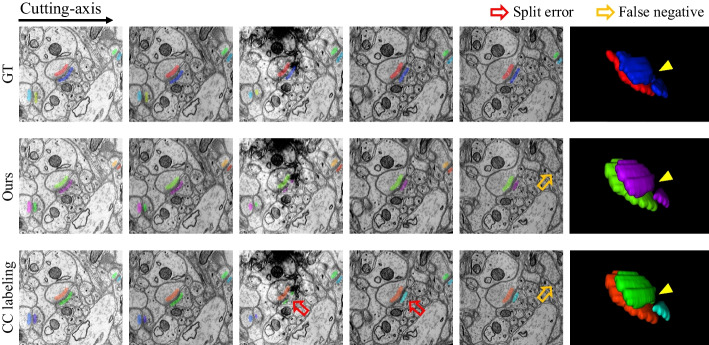
Qualitative comparison of synapse reconstruction performance from serial patches on Snemi dataset. From left to right are the sequential images along the cutting direction. The final column reveals the differences in 3D

Meanwhile, the quantitative result of ultrastructure reconstruction is recorded in Table 3. Unlike the Harris dataset, the ultrastructure is predicted by CNN to simulate the applicability and robustness of our method in real scenarios. From Table 3, the reconstruction performances differ greatly for the mitochondria and synapses. In most cases, the result of our algorithm and the proposed baseline are not inferior to those of CC labeling. Figure 12 gives a visualization of the differences. Specifically, the proposed method prevents a split error for synapse reconstruction because the imaging damage causes a local shift in the EM image. Noteworthy, the proposed baseline (i.e. Ours w/o neuron) is inferior to CC labeling for synapse reconstruction. Our analysis is due to sensitivity to edge weights, fine-tuning the bias hyper-parameter may improve the performance. Meanwhile, the joint optimization approach (i.e. Ours) outperforms CC labeling due to the neuron’s connectivity constraint.

Note that the error metric in Table 3 includes pixel-wise error and connection error. Particularly, the performance of mitochondria is significantly higher than that of synapses due to distinct regional features and shape invariance. We find the lower VI of synapse reconstruction mainly comes from pixel-wise errors, i.e., some synapses are not detected or inaccurately segmented boundaries, which is intuitively represented in Fig. 12. We can conclude that ultrastructure reconstruction is still subject to errors but does not affect the improvement of the neuron. This is based on the assumption that reconstructing subcellular structures is less difficult than reconstructing cellular structures. Moreover, our method only relies on the connection information extracted from the ultrastructure and is therefore insensitive to pixel-level errors. In other words, with the same image quality, we assume the connectivity information of the ultrastructure is more reliable than that of neurons, which is necessary to increase the confidence factor. We confirm this assumption in Fig. 11, where better results are obtained when the confidence factor of the ultrastructural connectivity features is higher than that of the neurons.

**Table 4:** The quantization performance of neuron reconstruction on the Snemi dataset

Methods	VI	VI-split	VI-merge	ARE
MC	0.6951	1.0520	0.3381	0.1297
GASPavg	0.6761	**0.9159**	0.4366	0.1339
MWS	0.7217	1.1003	0.3431	0.1406
MC w/ FM	0.7080	1.0681	0.3480	0.1316
MC w/ KL	0.6974	1.0543	0.3405	0.1299
Co-Clustering	0.6795	1.0134	0.3456	0.1259
Ours w/o mito	0.6864	1.0352	0.3375	0.1295
Ours w/o syn	0.6757	1.0125	0.3388	**0.1251**
Ours	**0.6701**	1.0034	**0.3369**	0.1253

**Table 5:** Comparison of the neuron reconstruction performance with and without ultrastructural mapping regions on Snemi dataset

Snemi					
	Methods	VI	Decrease	ARE	Decrease
w/	MC	0.3576		0.0735	
	Co-Clustering	0.3342	$-6.54\%$	0.0665	$-9.52\%$
	Ours	**0.3229**	$-9.70\%$	**0.0662**	$-9.93\%$
w/o	MC	0.8351		0.1297	
	Co-Clustering	0.8244	$-1.28\%$	0.1286	$-0.85\%$
	Ours	**0.8153**	$-2.37\%$	**0.1276**	−$1.62\%$

## Discussion and conclusion

In this paper, we investigate the joint optimization of ultrastructural and neuronal connectivity. We introduce the concept of the connectivity consensus based on biological domain knowledge for 3D agglomeration. We propose a joint graph partitioning model to determine the ultrastructural structural connectivity and membrane boundary connectivity. This method overcomes the limitations of using single connectivity cues. This optimization model has a single and well-defined mathematical objective, allows to produce the multiple structure reconstruction in a one optimization. We have demonstrated the advantage of joint optimization on several public datasets.

The goal of image analysis in connectomics is the 3D reconstruction of neural structures, and the agglomeration algorithm is a fundamental step. However, the reconstruction of neural structures in electron microscopic images is a very challenging task. The main factors affecting the reconstruction performance include imaging resolution, z-axis resolution, data volume, acquisition region and imaging quality, which result in reconstruction performance that may vary greatly between different data. Existing methods have the limitation that single connectivity features are not always reliable. The primary purpose of introducing connectivity consensus between different structures is to build a suitable optimization model that makes the reconstruction goals more consistent with biological plausible and domain knowledge.

Compared with voxel-based, superpixel-based methods are less sensitive to local defects at the membrane boundary. The proposed solution is robust to the over-segmentation issue. Specifically, if under-segmentation occurs in the neuron over-segment fragments, existing agglomeration algorithms are unable to correct such errors, so such errors are generally avoided at the watershed stage by adjusting hyper-parameters. If mitochondria occur under-segmented, this may only lead to performance degradation if two neurons in the plane to which the mitochondria originally belong are identified as one, because additional merge errors of neurons are introduced. However, as in Eqs. (2) and (3), we add the reconstruction confidence parameter $\lambda$ for different structures, which allows us to easily assign confidence levels based on the initial segmentation performance, as discussed in Fig. 11. In addition, we add 2D instance contour to the ultrastructure segmentation, which further reduces the possibility of under-segmentation. Note that the additional connectivity information used in this paper comes from mitochondria and synapses, which contain link-information and not-link-information that can be extended to other similar structures, such as cell bodies (same as mitochondria) and axon-dendrites (same as synapses). In the future, we plan to analyze the model’s performance for other biological structures and examine its generalization ability. Another important research direction is to extend the optimization model to integrate non-adjacent node relationships, i.e., long-range cues, such as lifted multicut algorithm.

## Data Availability

The public dataset supporting the findings is available at the following link. Harris dataset: https://neurodata.io/data/kharris15/ Snemi dataset: https://lichtman.rc.fas.harvard.edu/vast/AC3AC4Package.zip

## Abbreviations

EM: Electron microscopy
3D: Three-dimensional
CNN: Convolutional neural network
IoU: Intersection-over-union
RAG: Region adjacency graph
GAEC: Greedy additive edge contraction
VI: Variation of information
ARE: Adapted rand error
MC: Multicut
GASPavg: Agglomerative clustering with average linkage criteria
MWS: Mutex watershed
KL: Kernighan–Lin solver
FM: Fusion-Move solver
CC: Connected component
